# Using Appreciative Inquiry to explore the disruptive effect of COVID-19 on medical student trust in their Schools

**DOI:** 10.15694/mep.2020.000285.1

**Published:** 2020-12-18

**Authors:** Enjy Abouzeid, Nourhan Wasfy, Safaa El-Zoghby, Hani Atwa, Sherein Shalaby, Nancy Zaghloul, Nagwa Hegazy, Marwa Ahmed, Hebat Allah Amin, Mohamed Hany Shehata, Samar Ahmed

**Affiliations:** 1 Faculty of Medicine; 2 Faculty of Medicine; 3 Faculty of Medicine; 4 Faculty of Medicine; 5 Faculty of Medicine; 6 College of Medicine and Medical Sciences; 7 Faculty of Medicine

**Keywords:** Mistrust, Communication, Appreciative Inquiry, Extra-curricular activities, Medical Student, Mental Health.

## Abstract

This article was migrated. The article was not marked as recommended.

**Background**: Students anxiety due to the COVID-19 pandemic was expressed by some medical students in the form of anger and mistrust. This study aims to explore the reasons for mistrust between students and faculty among medical schools in Egypt that have flared during the pandemic.

**Methodology**: This is a three-phase exploratory qualitative study depending on thematic emergence from appreciative interviews (AI) sessions. ***Phase 1*** online Appreciative Inquiry (AI) session followed by Thematic content analysis. ***Phase 2*** The themes were approached by a smaller cohort of students using a design that relied mostly on the psychometric free association test. ***Phase 3*** The themes were tested on a larger number of students through an online survey.

**Results**: Students are revealed to be very well educated regarding contemporary medical education concepts. The most important factors from the student perspective were the presence of a well-designed assessment system aligned with the learning outcomes and teaching methodologies and the presence of extracurricular activities and soft skills, respectively. A balanced student life respecting their mental health was found important to increase trust.

**Conclusion**: A roadmap to breaking the mistrust must be planned on several pivots: curriculum structure, extracurricular life, communication strategies, and identifying student roles in their learning and decision-making.

## Introduction

Researchers defined trust as the act of sharing common cares and needs by people who value trust in their relationships. People value trust because they believed that they uphold each other’s truths and confidences ( Tschannen-Moran, 2014). Based on that definition, mutual trust between teachers and their students is markedly affected by the sense of caring and valuing expressed by the teachers and felt by the students, Byrk and Schneider (2002) argued that as trust ties increase between the students and teachers, more opportunities are there for teachers and students to positively affect student achievement levels.

Medical students in Egypt are experiencing unprecedented times, as they a re living in severe apprehension due to the state of uncertainty caused by the COVID-19 pandemic and its impact on their study ( Aker and Midik, 2020). Considering the new decisions, to sustain the educational process through online learning, students began to worry about their learning progress. The confusion raises several questions as to how long this state will last, how education will be continued without clinical rotations (which is considered the main component in any medical education curriculum), and whether they can cope with this new situation or not ( Rose, 2020).

This state of confusion, together with the continuous spread of the pandemic and the social distancing measures that were taken, result in a sense of stress and anxiety among the medical students ( Sahu, 2020). It was reported that public health emergencies have their impact on the psychological health of college students that could be elicited in the form of fear, anxiety, or worry ( Cao *et al*., 2020).

This anxiety was expressed by some medical students in the form of anger and refusal to use the online learning solution. They were uncertain about the appropriateness of these decisions and how this may affect their academic progression or future career. Additionally, this anxiety and confusion have created mistrust and hostile feelings towards the staff members and faculty administrators. This led the decision-makers, for example, to consider the students’ request to cancel the summative exams for the current academic year.

Taken all together, it is presumed that several factors may have contributed to the development of this mistrust and hostile attitude. Additionally, previous studies have documented a lack of communication between different levels in Egypt ( Shehata, *et al.*, 2020). Accordingly, this study aims to explore the reasons for mistrust between students and faculty among medical schools in Egypt that have flared during the COVID-19 pandemic.

## Methods

This is a three-phase exploratory qualitative study depending on thematic emergence from appreciative interviews (AI) sessions ( Smith, 2020).

A convenient sample size was calculated as 185medical students using online sample size calculator ( calculator.net, 2008) on a 5% margin of error and a 10% population proportion prediction.

### Phase 1

A purposive sample of 56 medical students from different medical schools in Egypt were invited to attend one online Appreciative Inquiry (AI) session through Zoom™ (Zoom Video Communications, Inc., San Jose, CA, USA). Thematic content analysis was done to the responses of the students in the interviews. Common themes were selected.

#### AI sessions outline:

The AI sessions focused on the process of describing the other lens by the students. Each session lasted for about 20 minutes and employed a double-barreled interview protocol. Students were divided into small groups of 4 to 5 students, and each group was assigned a faculty facilitator who interviewed them on their perceptions, viewpoints, and answers to a group of pivotal questions after the description of a hypothetical situation ( Table 1).

After each interview, the students reconvened in one large group together with the faculty facilitators.

**Table 1.** Outlines of AI interviews with the students

Scenario	Guiding questions	Focus
***Interview 1:***		
Imagine a time when you felt really secure and happy in your school.	What was happening?Why do you think this was a successful encounter?What are the factors that led to this success?	Past Factors
*Student reconvening, with thematic deduction from interviews*		
***Interview 2:***		
Imagine it is now 2025. You are in an amazing teaching environment. You are at your best. You feel extremely secure and happy in your school.	What is happening? (Be very graphic) What are the factors that lead to this success?	Future Factors
*Student reconvening and an expression of how you feel reflecting on the process*		
Tell us the story of your success. Imagine you are in a television interview as a Dean of a medical school.	How did it happen?What changes did you need to do to reach this situation?	Action Steps
*Student reconvening, with thematic deduction from interviews*		

### Phase 2

The themes that emerged from the interviews were approached by a smaller cohort of students using a design that relied mostly on the psychometric free association test.

#### Association Test:

A test used in psychology to study the organization of mental life, with special reference to thecognitive connections that underlie perception and meaning, memory, language, reasoning, and motivation. In the free-association test, the subject is told to state the first word that comes to mind in response to a stated word, concept, or other stimuli. In “controlled association,” relation may be prescribed between the stimulus and the response (e.g., the subject may be asked to give opposites). Though more complex analyses may be used for special purposes, the reaction time for each response and the words the subject gives are the basic data provided by the test ( Rogers, 2020).

The themes that emerged from the first interview were grouped in non-coherent yet thematically attached groups of words. These groups were mapped on an online blank wall board that can be used by invited participants to collaborate in collecting ideas, brainstorming, and sharing information. Students were first sensitized to the positive thinking mode using a 20-minute picture-based presentation introducing the concept of AI. Students were then asked to read each box and identify the first word (academic story) that comes to his/her mind. They were then coached into star rating each box of themes from 0 to 5 based on the sense of urgency they felt associated with it. Students were instructed to think of a more positive approach when handling the themes. The top-rated theme boxes were chosen and smaller groups of students were asked in a smaller group to tell stories that come to their mind when reading those themes and to highlight the factors that lead to this memory being an important one that they still have till the moment.

### Phase 3

The themes that emerged from the interviews were tested on a larger number of students through an onlinesurvey disseminated to 279 medical students (exceeding the calculated sample size) in Egypt through various platforms. The survey aimed to assess the frequency and prioritize the causes of students’ trust in their medical schools. The questionnaire was developed by the authors according to the data analysis of the interviews. It was developed on Google forms; https://forms.gle/i2iMBwqFA4ghL9677 (Supplementary file 1).

#### Ethical considerations

The participants were informed about the purpose of the study and its relevance to the field of medical education. Only those who agreed to be involved in the study were included. Participants’ names and universities were kept highly confidential. Any information the participants included in the questionnaire or interviews were treated with confidentiality. However, participants were given the right to withdraw from the study at any time without any consequences.

## Results/Analysis

The core of the AI session was to allow students to picture a perfect future where they felt safe and trustful in their learning environment. The themes that emerged from the student pictured dreams in Phase 3 of the AI session were collated into themes and were categorized as follows:

### A. Curricular Adaptations and Innovations in Learning Strategies:

The students emphasized that the curriculum contents and resources should be continuously updated and modified, to let them acquire the general practitioner’s core competencies. They identified that linking theoretical parts of the curriculum with clinical practice settings from the first study year was identified as one of the empirical factors for engagement.

The student responsibility for selection of learning resources and omission of unified resources was also mentioned as a factor related to their sense of engagement.

According to students’ perspectives, knowledge is available in books and the internet, and medical schools should provide experiences that are beyond books and other resources. A quote by one student is:

“.. there are important skills that should be represented in the curriculum, such as communication skills, time management, entrepreneur thinking, and public speaking or presentation skills. Teaching communication skills is of particular importancefor being a successful doctor”.

Availability of different sources for psychomotor skills training as skills labs, simulation rooms, and early patient encounters was highly noted by the students.

Students preferred integrated teaching sessions organized in such a way that it cuts across subject-matter lines, bringing together various aspects of the topic into a meaningful association. These sessions offer enjoyable learning experiences, leading to students’ satisfaction with the educational environment. They recommended a proper arrangement of the content and media to avoid repetition and to facilitate the constructivist approach of learning through building on the previous knowledge.

Integration of technology in medical education was highlighted by students. One student stated:

“We should have simulation rooms for practicing surgical and laparoscopic operations for instance, not just traditional skills labs, with allowing for appointment system where each student could easily choose to be trained in the area of his interest”.

Some students suggested the use of high-fidelity simulation as virtual reality, robotic surgery, and laparoscopy. Regarding the advantages of simulation, one of the students mentioned:

“Trying several times allows us to get confidence before being exposed to real patients”.

They added the need for continuous update of the clinical training to include any new application or techniques as they may contribute to telehealth and help in health education and health promotion.

The students also offered solutions for the financial support for all these innovative techniques, like the adoption of sponsors who can afford the expenses of the implementation of these techniques.

The students raised an important point regarding active learning. One student commented:

“No more didactic lectures are needed; instead, small group discussions with session moderators with triggering questions that elicit creativity and critical thinking and help us integrate the learned sciences together”.

They added that this will help them acquire the skills of teamwork and train them to manage its conflicts, preparing them to face the real life.

The students pointed out to the importance of working as a part of research teams which include staff and studentsto increase their research skills. Additionally, they recommend that their schools support the national and international publications of their research.

### B. Students as Active Stakeholders:

The students recommend the need of being independent learners and take control of their own learning. One student mentioned:

″I prefer replacing spoon-feeding teaching strategies with other active learning strategies that target more than just simple recall of facts″.

They expressed appreciation towards learning strategies that foster students’ engagement and competitions as problem-based learning, team-based learning, evidence-based learning, and flipped classrooms. One student commented on problem-based learning as:

“.. this strategy has lifted up my college experience”.

Many students expressed that each moment of learning via such interactive strategies is so capturing and motivating. All these strategies allow the students to be in close and direct contact with the teachers, who facilitate their learning. Interactive sessions facilitate communication and exchange of ideas. Moreover, it helped the students to engage and gave them the feeling of ownership of their learning.

#### According to one of the students:

“Allowing the students to participate, either in the form of explanation or application of knowledge, is the best way for ensuring students learning”.

#### Another student highlighted the importance of treating students as adult learners:

“I need to take the responsibility of my learning. I want to be the pacemaker”.

The students highlighted that one of the main factors for their engagement and motivation is being actively involved in the learning process. Active involvement can take several forms. One of these forms is the use of student feedback and evaluation of the educational activities to improve the learning process. Another form is the modification of the learning activities to be student-centered and involved active discussions.

Sharing students’ ideas and being able to communicate these ideas were important points highlighted in the discussion. Students need space and a chance to trace their passion and dreams.

### C. Student Life:

Extracurricular activities can help in the personal development of the learners and facilitate the building of a community between the students. Examples of extracurricular activities are community participation and voluntary work. The students believe that they should be fostered and included in the student assessment plan.

The students also disclosed the significance of mental health support and guidance for the students.

A friendly campus and the availability of different facilities that may help the students to engage with the place and spend a high-quality time in it will increase their satisfaction, engagement, and academic achievement. This development should also include the infrastructure and safety measures for the students and doctors through legislation to protect their rights. Also, the campus should include computers, internet connection, and study landscapes. A reward system should be settled to motivate students who achieve success in their extracurricular activities.

#### One student stated:

“I should have time to be a doctor and, in the same time, live my life!”.

### D. Relationship with Administration:

The students appreciate it when their voices are heard. They prefer to be involved in the planning, implementation, and evaluation phases of different educational activities (student-centeredness). They suggested the development of a medical education quality committee with student representatives.

The administration should provide early and continuous student orientation about each step and milestone in their learning experience. This orientation will help the students to be responsible and aware of what is expected of them. Continuous and open communication with the administration is requested by the students, who recommend a continuous evaluation with the involvement of the students’ opinions and communicate with them the evaluation results.

They also referred to the free open discussions with the school’s administration that allow for expressing their points of view and their capability to point out their opinionssafely without being humiliated.

#### One student reported:

“In our medical school, not all the faculty members have the ability to facilitate learning, although being competent physicians .. a matter that should be tackled by helping them acquire such skills early in the undergraduate life”.

They need all their feedback to be considered, with real investigation of root causes of problems faced and implementing real corrective actions as needed for continuous quality improvement. They also mentioned that transparency is the key to building mutual trust between them and their teachers.

#### E. The Progressive Vision of the University:

**Figure 1.**
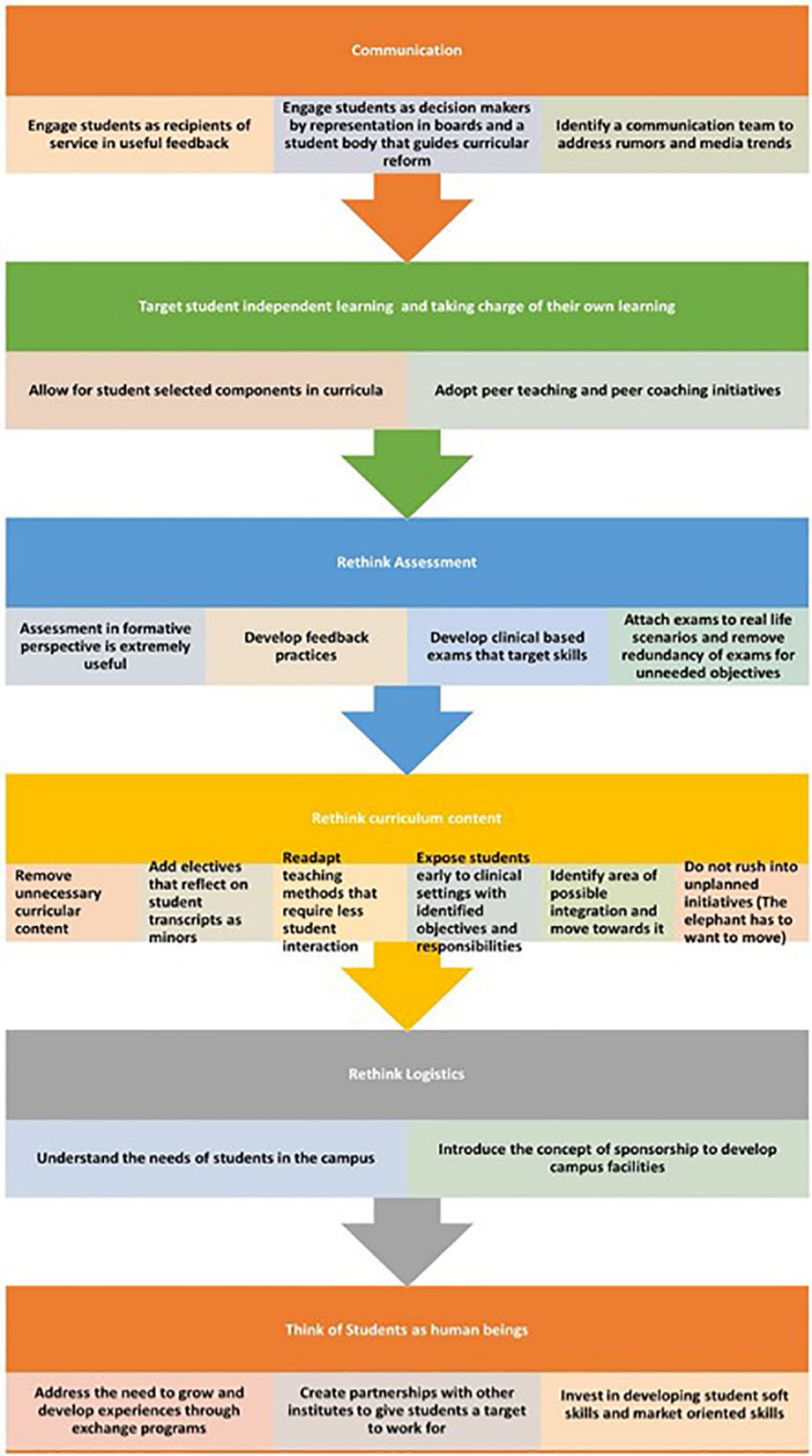
Social Accountability triad (original)

Medical schools should support students with various opportunities for national, regional, and international collaboration and exchange. According to one of the students:

“Schools should develop social accountability with three pillars: research, services, and clinical practice .. and the student is in the center of all these concepts” as shown in Figure 1.

#### Another student added:

“It is important that our school seeks partnerships with highly-ranked institutions and reach out for community services”.

Medical schools should be aware of the surrounding continuously changing needs of the community with being in one line with the strategic plan of the government.

#### One student mentioned:

“Coinciding with developing the National Insurance System, we need to focus more on graduating competent general practitioners to serve their community; a matter that should be considered in the vision of our medical schools now”

The students indicated the importance of creating educational facilities in all hospitals whether affiliated to the Ministry of Higher Education or Ministry of Health, allowing for more job opportunities for the graduated physicians without the need for the traditional competitive system for physicians’ allocation, with more deep collaboration between both ministries.

### F. How the School Views Student Assessment:

The students suggested that medical schools should reconsider the attendance grades and focus more on the active attendance of the students and the way they actively participate in interactive learning sessions rather than their mere presence in such sessions.

The students highlighted the importance of standardization of oral exams. They believe that using predefined questions in the exams, viva cards, and checklists for evaluation will ensure that standardization. One student mentioned:

“It doesn’t matter who will be the examiner in our oral exams if they are standardized”

They also prefer the objective test formats, such as multiple-choice questions, rather than subjective formats, such as the essay questions.

The students prefer a radical change in the assessment scheme in which higher-level assessment methods such as OSCE, WPBA, and other objective clinical exams should account for the highest percentage of the final grade. Thus, they suggest a shift from exam-based assessment to competency-based assessment.

### G. Role of Staff Members and Mentorship:

Student advisor or mentor may help the new students in understanding the shift from the high school education to the university level. Also, it may help the senior students in career choice. Mentors can be staff members, senior students, or even peers.

The students reflected that the support of their mentors has guided them to understand the current situation in the COVID-19 pandemic crisis and reduced their panic and stress. They also added that mentors should be responsive, cooperative, and elaborative. Mentors should foster continuous communication and provide clear explanations and close relationships with the students.

The students place emphasis on the importance of the presence of a mentor who could facilitate the learning process; a one they could return to directly on having any inquiries regarding their research, clinical training, or their progress in general with encouraging and inspiring them for more progress.

The presence of sympathetic staff members who concern about the students’ social and psychological aspects besides the academic one is repeatedly mentioned by them as the main factor of feeling secure at school. They also mentioned that the presence of reachable staff members, for instance through office hours or prescheduled online meetings, will be a helping tool for the continuous learning process.

The students believed in the important role of continuous faculty development and selection of the faculty according to various standards not only their scientific level. Furthermore, qualified university leaders should be assigned to significant positions and responsibilities.

Teachers can shape the students’ learning environment. Accordingly, they should foster student motivation and engagement using innovative teaching approaches.

Testing on a Representative Sample of Students

Two hundred seventy-nine medical students responded to the survey. Students were from 9 different medical schools in Egypt.

**Figure 2.**
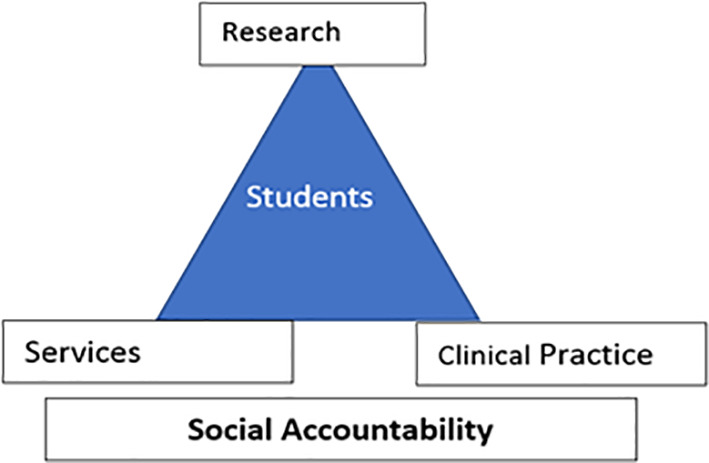
Top 10 factors affecting students’ trust in their mecial schools

The top ten factors affecting the students’ trust are highlighted in Figure 2.

Table 2 highlights the responses of the medical students to the factors affecting their trust in their medical schools. The factors are divided into 7 domains including student assessment, curricular adaptations, relationship with the administration, students as important stakeholders, progressive vision of the university, relationship with the administration, and student life.

Regarding student assessment and curricular adaptation, the most important factors from the student perspective were the presence of a well-designed assessment system aligned with the learning outcomes and teaching methodologies and the presence of extracurricular activities and soft skills, respectively.

The ability of the medical school to help the students in having a balanced student life respecting their mental health was the most important perceived factor in the student life domain.

**Table 2.** Frequency of perceived factors increasing student trust in their medical schools

Domain	Disagree		Neutral		Agree	
	N	%	N	%	N	%
Regarding **“ *STUDENT ASSESSMENT*”**, the degree of trust in my medical school will increase when it ...						
adopts competency-based projects as a method of student assessment	51	18%	63	23%	165	60%
uses assignments as a well-designed method to assess the student knowledge and competencies	55	20%	48	17%	176	63%
uses a flexible pass/fail (not grades) system in the transition years	79	28%	42	15%	158	57%
uses fair, competency-based portfolios plus an exam-based system of evaluation in the final years	41	15%	55	20%	183	66%
has a more structured, objective system for oral exams	74	27%	48	17%	157	56%
has a well-designed assessment system aligned with the learning outcomes and teaching methodologies	13	5%	37	13%	229	82%
Regarding **“ *CURRICULAR ADAPTATIONS*”**, the degree of trust in my medical school will increase when it ...						
conducts extracurricular activities and soft skills	30	11%	38	14%	211	75%
allows students to choose more than one major	29	10%	53	19%	197	71%
has non-medical pass/fail courses in its curricula	84	30%	77	28%	118	42%
has more applied practical sessions in basic sciences	14	5%	33	12%	232	83%
has well-structured, non-redundant curricula with early clinical exposure	12	4%	40	14%	227	81%
has various student assessment methodologies for continuous assessment during the module/course	26	10%	48	17%	205	73%
has a platform for medical students with quality educational material	8	3%	41	15%	230	82%
Regarding **“ *STUDENTS AS IMPORTANT STAKEHOLDERS*”**, the degree of trust in my medical school will increase when it ...						
addresses the role of peer (student) mentors in the educational process and psychological support of a medical student	20	8%	41	15%	218	79%
has actual students’ representation in decision-making	15	5%	42	15%	222	80%
creates students’ taskforce that contributes to the development of the faculty and community	25	9%	53	19%	201	72%
establishes a platform for medical students to express themselves and have their voices reach the decision-makers	14	5%	32	11%	233	84%
Regarding **“ *PROGRESSIVE VISION OF THE UNIVERSITY*”**, the degree of trust in my medical school will increase when it ...						
applies admission tests for joining medical schools	67	24%	62	22%	150	53%
allows student contribution to institutional charity work	19	7%	46	16%	214	76%
enforces the role of staff mentors in the wellbeing of the medical student	24	9%	49	18%	206	74%
conducts public speaking and presentation courses/activities included in our portfolio	38	14%	46	16%	195	70%
is an interdisciplinary institution (allows for multiple specialties; medical and non-medical)	50	18%	65	23%	164	59%
has partnerships with highly ranked national and international medical schools	9	3%	31	11%	239	86%
Regarding **“ *RELATIONSHIP WITH THE ADMINISTRATION*”**, the degree of trust in my medical school will increase when it ...						
enforces mutual student-administration and student-staff trust	17	6%	42	15%	220	79%
has administrative accountability and transparency	16	5%	44	16%	219	78%
appoints highly qualified leaders	14	5%	25	9%	240	86%
administration and staff members acknowledge the presence of defects and work for change	7	2%	27	10%	245	88%
conducts actual faculty development programs	8	3%	26	9%	245	88%
has community service programs	17	6%	38	14%	224	81%
Regarding **“ *STUDENT LIFE*”**, the degree of trust in my medical school will increase when it ...
helps me have a balanced student life respecting my mental health	7	3%	23	8%	249	89%
has a platform for students’ mental, psychological, and academic support	7	3%	16	6%	256	92%
has a beautiful campus with well-designed studying landscapes	12	4%	21	8%	246	88%
collaborates with sponsors for upgrading its infrastructure (including hospitals)	7	2%	17	6%	255	92%
has high fidelity simulation labs and virtual reality technologies	6	2%	18	6%	255	91%
has a constructive multisource feedback system	7	2%	20	7%	252	90%
has advanced training courses for students and staff members	7	3%	19	7%	253	91%
has legislation for protecting doctors working in teaching hospitals	9	3%	22	8%	248	89%
has students and staff exchange programs	10	4%	32	11%	237	85%

## Discussion

Effective leadership requires receiving feedback from the stakeholders for proper management of crises. Students are significant stakeholders in the educational process, and they must be seen and heard for highly relevant feedback. However, gaining the students trust is not only through communication because students are now very well-educated, have vision, and they can critique all the strategies. Accordingly, strategizing curriculum reform with involvement of the students is highly recommended for the development of medical education and practice in Egypt.

Findings in our study are unique in the sense that the degree of trust of the students in their universities is attributed to more factors than simply the traditional open communication theory. Students have identified both qualitatively and quantitatively that they are in continuous evaluation for their school’s educational practices, curricular reform activities, assessment decisions, and social accountability role, this is in agreement with Kumar and Jain (2018). This is a finding that we can attribute to the open nature of information at the moment and the effect of student bodies that exist within medical schools and that have a great role in educating students about their own education ( Bruffee, 1999). The results of the current study revealed important points regarding the students. Firstly, they are very well-educated regardingcontemporary medical education concepts. Secondly, they are not exam-oriented students, but they are targeting the international work standards. Finally, they want to build a mutual relationship with the university and community as well.

Overall, students are seeking fairness and involvement. Fairness includes equal and appropriate clinical training, standardized assessment methods, legislation for protecting doctors, and fair work opportunities. Involvement includes student-centered learning strategies, interactive sessions, and usingtheir feedback in the continuous learning process. In line, the use of high-fidelity technology in teaching and clinical training was selected among the top-rated factors to possibly increase student trust in their universities. Simulation may help in the development of trust as it may improve the students’ conceptual understandings and training for future practice ( Srisawasdi and Sornkatha, 2014) and apply their learning in a safe environment before patient exposure, as simulation is one of the strategies which are suggested and used to reduce stress and anxiety in clinical student education ( Mohammadi *et al.*, 2019). This is also justified by the era we are living in post COVID-19 and the stress that is put on the shoulders of students regarding the opportunities that still exist for them to learn clinical skills despite the social distancing ( Shehata *et al.*, 2020).

Student work-life balance came high on the list of factors. This was seen also in the need for a beautiful campus and the need to have a psychological and social support system in the school. This is a need for the students that came in agreement with previous studies which stated that if the educational institute provides a healthy environment, students will be more comfortable, satisfied, and able to interact with each other and their teachers ( Khurshid and Arshad, 2012; Tarquinio, 2016).

The students also highlighted the importance of mental health support that may affect their performance and satisfaction. Similarly, studies have highlighted that stress reduction and management are effective for the health and well-being of students who will eventually become the future doctors taking care of physical and mental wellbeing of other ( Huynh *et al.*, 2020; Kötter *et al*., 2019). Conley, Kirsch, Dickson and Bryant (2014) reported that students’ involvement in extracurricular activities can improve their mental health which is confirmed by our students’ opinion who believed that involvement in extracurricular activities may shape their personality and develop transferable skills. But it is important to consider enough time for these extracurricular activities in the curriculum planning as the lack of time is the most frequently mentioned barrier for student engagement ( Almasry *et al.*, 2017).

The use of standardized, structured assessment methods was selected as one of the factors affecting students’ trust. The students agreed that those methods might help in building trust with their organization as they ensure fairness and prepare them for their roles as doctors. Trust will be developed when students are given equitable opportunities to demonstrate what they have mastered. In addition, students need to feel comfortable that assessment is reflecting a real picture for their competencies and capabilities. This is in consistency with the medical curriculum reform in Egypt that obligated the use of standardized, structured assessment methods that have high psychometric measures. Likewise, the Egyptian National Academic Reference Standards (NARS) emphasizes that universities should make all the efforts to establish an assessment system that utilizes a variety of methods. This requires the use of objective questions in addition to modified essay questions, problem solving exercises, and case studies in written exams. Similarly, universities must ensure that assessment of clinical and practical skills encompasses tools that allow the coverage of a wide variety of required competencies ( NAQAAE, 2017).

Additionally, traditional learning strategies were no longer satisfactory for the new century students. They claimed that experiencing interactive, student-centered learning sessions provides them with confidence and prepare them in an authentic way. Moreover, the use of team-based learning andproblem-based learning improves their engagement and knowledge acquisition. This agrees with Smeby *et al.* (2020) who reported that these strategies were rated significantly higher than traditional lectures on making difficult material comprehensible, increasing student engagement, and giving the students feedback on their own knowledge.

The students are expecting a structured process for the recruitment and promotion of staff members. This process should ensure high qualifications of the staff as subject matter experts and medical educators. This will help the staff understand their role that in turn will reflect on both the students and learning experience. They suggested a continuous faculty development to cope with the transforming world. This is in congruence with Ahmed *et al.* (2020) who reported that the recent chaotic transition to virtual platforms lifted the lid off a current need for faculty development that was not identified before. This was also confirmed by other studies that reported that faculty is considered be the backbone of any institution and their training and development are necessary for progressive growth of an educational organization ( Ahmed *et al*., 2020).

The relationship between the students and the teaching staff is very important for the success of the students and the university, and to facilitate this relationship approachability is very important. The students requested continuous guidance in the form of mentorship programs, either staff or peer mentoring. The students confirmed that they need guidance during the different phases of their study. Junior students need guidance for smooth transition from school learning to university context. Peer mentoring can be more helpful for these junior students who may be more comfortable raising areas of uncertainty with senior students, and a subsequent increase in knowledge, skills, and confidence can enhance their future interactions with clinicians, ( Choudhury *et al.*, 2014; Nimmons *et al.*, 2019) while senior students need guidance for career choice ( Kollias *et al.*, 2010) as early mentoring can offer students an insight into what it is like to work in that specialty and challenges preconceptions they may have ( Farrell *et al.*, 2013). Thiswould increase confidence, self-perceived preparedness for starting work as doctors, and a reduction in the performance gap ( Dalgaty *et al.*, 2017).

Additionally, how the school leadership are chosen and thus how this will impact progressive decisions made by the school like partnerships with external bodies andstudent exchange opportunities were highly scored factors in the students list. These factors may allow more transparency and active open channels of communication that may help the students to accept decisions and in turn decrease the student mistrust in the organization. Additionally, this leadership may allow their representation in the different committees and to play an active role in the decision-making process using their feedback in program evaluation. Despite these measures may be adopted in some schools according to the recommendations of the international and national reference standards for quality improvement, it seems that the measures are not enough or not reachable by all students. However, there is evidence that students are no longer thinking about their schools as a phase that ends with the end of their study years. They understand the impact of the opportunities they can get inside the school and how these can impact their whole future ( Engel, Gibson and Gatalica, 2019).

## Conclusion

Medical students in Egyptian universities are no different than other schools in other countries. Students are suffering from a large amount of mistrust towards their universities, administration, and faculty. This is the heritage of years of failed communication. A roadmap to breaking the mistrust must be planned on severalpivots; the curriculum structure, extracurricular life, communication strategies, and identifying student roles in their learning and in decision making.

## Take Home Messages

To regain students trust, universities need to:

• Engage students as decision makers.
• Provide opportunities for student independent learning.
• Redesign assessment system to become more valid, less redundant and better balanced in terms of formative and summative.
• Rethink curriculum content by removing unnecessary content, add electives, re-adapt teaching methods that require less student interaction, ensure early clinical exposure, identify area of possible integration and move towards it.
• Rethink Logistics with better understanding of the needs of students in campus.
• Think of Students as human beings who need to grow and develop experiences.
• Invest in developing student soft skills and market-oriented skills.

## Notes On Contributors

**Enjy Abouzeid M.:** MSc, Medical Doctorate, University of Leeds & FOMSCU. She is a Lecturer in the Medical Education Department. She is working as Head of the exam committee (MCQs) for the undergraduate phase. Also, she is a Vice President of the assessment and evaluation unit in the Faculty of Medicine, Suez Canal University, FOMSCU. She is a Faculty in the Diploma of Health Professions Education program (DHPE) and an Online discussion coordinator of the Joint Master of Health Professions Education between Maastricht and Suez Canal Universities (JMHPE) and DHPE. ORCID ID: https://orcid.org/0000-0002-9431-6019

**Nourhan F. Wasfy:** MSc, Medical doctorate **.** She is a Lecturer of Medical Education in the Medical Education Department. she is Head of the Portfolio committee for undergraduate phase 1, member of the assessment and evaluation unit, member of Quality Assurance unit, Faculty of Medicine, Suez Canal University (FOM-SCU). Coordinator of the Diploma of Health Professions Education program (DHPE)-FOM-SCU. ORCID ID: https://orcid.org/0000-0002-2896-9142

**Safaa Mohammed El-Zoghby:** MD. She is a Lecturer of Family Medicine, and Field training coordinator for the 1st year in Faculty of Medicine, Suez Canal University, Ismailia, Egypt. ORCID ID: https://orcid.org/0000-0003-3978-5565

**Hani Atwa:** MD, MHPE, PhD.He is an Associate Professor of Medical Education at Suez Canal University in Egypt. ORCID ID: https://orcid.org/0000-0002-0099-4100

**Sherein Abdelhamid Shalaby:** MSc, MD, FAIMER fellow 2020. She is a Professor of Pediatrics, Head of Pediatrics Department, Director of the medical education unit, member of the Steering Committee for the MBBCh program, and member in the Curriculum and Education Committee and the research plan committee, Faculty of Medicine, Helwan University (FMHU).

**Nancy M. Zaghloul:** MSc, MD. She is an Assistant professor of Forensic Medicine and Clinical Toxicology Department. Vice director for the Quality Assurance Unit, Head of student activities & Research committee, Academic years Control president, E-learning coordinator, and member of medical Education Centre (MEDC), Faculty of Medicine, Misr University for Science and Technology. ORCID ID: https://orcid.org/0000-0002-1596-7367

**Nagwa N. Hegazy:** MSc, MD, DHPE, FAIMER Fellow. Assistant professor of Family Medicine. Director of the Medical Education and Human Resources Development Center, Faculty of Medicine, Menoufia University (MU). Head of the digital transformation committee, member in the AKT and Board member in the Egyptian fellowship of Family Medicine. ORCID ID: https://orcid.org/0000-0001-9470-5105

**Marwa M. Ahmed:** MSc., MD, MRCGP (Int.), FAIMER Fellow 2019. She is an Assistant Professor of Family Medicine and Acting head of Family Medicine Department, Faculty of Medicine, Cairo University, Egypt. Board member of Egyptian fellowship of Family Medicine. ORCID ID: https://orcid.org/0000-0002-5461-7861

**Hebat Allah A. Amin:** MSc, MD, AICPD, FAIMER fellow 2020. She is a lecturer of Histopathology, the Academic Co-chair of the Steering Committee for the MBBCh program, phase I coordinator, Head of the E-Learning Committee, and member in the exam Committee and the medical education unit, Faculty of Medicine, Helwan University (FMHU). ORCID ID: https://orcid.org/0000-0003-3311-4840

**Mohamed H. Shehata:** MSc, MD, MHPE, FAIMER Fellow. He is a Professor of Family Medicine - AGU. Faculty at EMR Regional FAIMER Institute. He founded the Medical Education Unit at Helwan University. Worked as an educational consultant in the Egyptian Fellowship. In Suez Canal University he led the school’s teams of field training, Clinical teaching, and OSCE. ORCID ID: https://orcid.org/0000-0001-7069-9329

**Samar A. Ahmed:** Medical Doctorate, MHPE, FAIMER Fellow, UNESCO TOT, Full professor in Forensic Medicine Ain Shams University, Director of ASU-MENA-FRI. She has a wide experience in project management and proposal writing after being a part of the Ministry of Higher Education EU project team for quite some time. She held many educational positions as a director of the quality assurance unit and the Director of the education development unit in more than one university. ORCID ID: https://orcid.org/0000-0001-8119-9258
