# Supplementary material for: Using Appreciative Inquiry to explore the effect of COVID-19 on student trust: medical students’ perspective
Source: MedEdPublish (2016). 2021 Sep 23;9:285. Originally published 2020 Dec 18. [Version 2] doi: 10.15694/mep.2020.000285.2 (PMC10697531; doi:10.15694/mep.2020.000285.2)
Supplement: Peer review history

# Reviewer report: Reviewer response for version 2

**DOI:** 10.21956/mep.20289.r31506

**Published:** 2021-10-08

**Authors:** Richard Hays

**Affiliations:** 1 James Cook University

This review has been migrated. The reviewer awarded 4 stars out of 5 The authors have addressed the comments of the reviewers of the previous version. The report provides an interesting window on how things may be changing as younger people embark on medical careers. Not so long ago, trust was in established institutions and professional norms. Students would accept (at least outwardly) the curriculum and teaching methods provided. Student engagement has long been recognised as important to learning, but this adds the trust dimension. It would be interesting to gain a multi-cultural, international perspective on the issues. The article is a worthwhile read for all involved in curriculum development and student support.

Reviewer Expertise:

NA

No decision status is available

# Reviewer report: Reviewer response for version 2

**DOI:** 10.21956/mep.20289.r31507

**Published:** 2021-10-01

**Authors:** Ken Masters

**Affiliations:** 1 Sultan Qaboos University

This review has been migrated. The reviewer awarded 4 stars out of 5 I am pleased to see that the authors have addressed the main concerns that I had with Version 1 of their article, and have produced a great piece that is a valuable contribution to the field.

Reviewer Expertise:

NA

No decision status is available

# Reviewer report: Reviewer response for version 1

**DOI:** 10.21956/mep.19146.r27683

**Published:** 2021-04-26

**Authors:** Ken Masters

**Affiliations:** 1 Sultan Qaboos University

This review has been migrated. The reviewer awarded 2 stars out of 5 The article deals with the disruptive effect of COVID-19 on medical student trust in their Schools.The paper begins by identifying the issue of trust, and the impact that COVID-19 has had on medical students' trust of their medical schools in Egypt. The article then aims to explore the reasons for mistrust.It is pleasing to see that the authors are attempting to use Appreciative Inquiry, a methodology that is not widely used in medical education. As the other reviewers have noted, however, there are some problems with the manner in which it has been implemented, and I trust that the authors will be correcting their assumptions, phrasing and descriptions in Version 2 of the paper. (I will not repeat them here, as the points have already been made).In the Results reporting, in addition to what the others have said, I would like to see a little more about the theming process. Who did it, the process of verification, software used, etc.I look forward to version 2 of the paper in which these issues are addressed.

Reviewer Expertise:

NA

No decision status is available

# Reviewer report: Reviewer response for version 1

**DOI:** 10.21956/mep.19146.r27684

**Published:** 2021-02-20

**Authors:** Megan Anakin

**Affiliations:** 1 University of Otago

This review has been migrated. The reviewer awarded 2 stars out of 5 My review is focused on ways to improve this article. In the introduction section, please provide supporting references for the claims made in the paragraph beginning ‘This anxiety was …’ In the final paragraph in the introduction section, please remind the reader what the several factors are by listing them and explain how a lack of communication between different levels might impact these factors and relate to the study’s aim.Please provide a scholarly reference rather than a link to a commercial website that sells products to support the Appreciative Inquiry interview approach used in this study. Please also revise the description of the study design to state that it uses mixed methods.Please consider removing the statement beginning ‘A convenient sample size …’ because it does not apply to qualitative study sampling and it contradicts the statement about purposive sampling in the next sentence.I agree with reviewer Mary Higgins, please provide a reference for the thematic content analysis methods used and explain the steps used by the research team to arrive to the themes presented in the results section. Please consider reading: Jacqueline Low (2019) A Pragmatic Definition of the Concept of Theoretical Saturation, Sociological Focus, 52:2, 131-139, DOI: 10.1080/00380237.2018.1544514. Please specify ‘other lens’ and ‘double-barrelled interview protocol’ used in the AI sessions, and ‘non-coherent yet thematically attached groups of words’ and ‘to think of a more positive approach when handling the themes’ in the interview analysis procedure. For phase 3 of the study, please explain how these data were analysed and results were used to address the aim of the study. When reporting themes from a qualitative analysis, please either give the participants pseudonyms or random alphanumerical identity codes (such as P1, P2, …) so the reader can see that the quotations do not all come from one student. For suggestions about presenting quotations in the results section: Lingard, L., 2019. Beyond the default colon: effective use of quotes in qualitative research. Perspectives on medical education, 8(6), pp.360-364. https://link.springer.com/article/10.1007/s40037-019-00550-7In the discussion section, please explain and provide a reference to support the phrase “simply the traditional open communication theory’ because this theory is not introduced to the reader in the introduction or here. Please check the accuracy of statements in the discussion section. For example, please consider revising the sentence beginning ‘Students have identified both qualitatively and quantitatively’ with something like ‘Qualitative and quantitative findings can be interpreted to support students views that ….’A discussion of the study’s methodological limitations and possible future studies are missing in the discussion section. To address this need, please consider: Lingard, L., 2015. The art of limitations. Perspectives on Medical Education, 4(3), pp.136-137.I would be very happy to read and review a revised version of this article.

Reviewer Expertise:

NA

No decision status is available

# Reviewer report: Reviewer response for version 1

**DOI:** 10.21956/mep.19146.r27685

**Published:** 2020-12-21

**Authors:** Mary Higgins

**Affiliations:** 1 University College Dublin, National Maternity Hospital

This review has been migrated. The reviewer awarded 3 stars out of 5 Thank you for this interesting paper, with findings that I consider to be universal!I do find the title a little misleading as Appreciative Inquiry is based on the premise of what is good rather than not, so why the students do not trust the system seems contrary to this. Can you clarify the sample size decision? For qualitative research it usually depends on when thematic saturation occurs so this is an interesting way, but needs further information to explain. You may also be interested in the work of Braun and Clarke, who described thematic analysis and do not believe that themes "emerge" but that they are developed.

Reviewer Expertise:

NA

No decision status is available
